# Pulmonary Disease due to *Mycobacterium tuberculosis* in a Horse: Zoonotic Concerns and Limitations of Antemortem Testing

**DOI:** 10.1155/2012/642145

**Published:** 2012-04-09

**Authors:** Konstantin P. Lyashchenko, Rena Greenwald, Javan Esfandiari, Alexis Lecu, W. Ray Waters, Horst Posthaus, Thomas Bodmer, Jean-Paul Janssens, Fabio Aloisio, Claudia Graubner, Eléonore Grosclaude, Alessandra Piersigilli, Irene Schiller

**Affiliations:** ^1^Research and Development Department, Chembio Diagnostic Systems, Inc., 3661 Horseblock Road, Medford, NY 11763, USA; ^2^Museum National d'Histoire Naturelle Department, Paris Zoo, 53 Avenue de Saint-Maurice, 75012 Paris, France; ^3^Infectious Bacterial Diseases Research Unit, National Animal Disease Center, Ames, IA 50010, USA; ^4^Institute of Animal Pathology, Vetsuisse Faculty, University of Bern, P.O. Box 8466, 3001 Bern, Switzerland; ^5^Institute for Infectious Diseases, Medical Faculty, University of Bern, 3010 Bern, Switzerland; ^6^Division of Pulmonary Diseases, Geneva University Hospitals, 1211 Geneva, Switzerland; ^7^Equine Clinic, Vetsuisse Faculty, University of Bern, P.O. Box 8466, 3001 Bern, Switzerland; ^8^Cantonal Veterinary Office Geneva, Quai Ernest-Ansermet 22, Plainpalais P.O. Box 76, 1211 Geneva 4, Switzerland; ^9^Monitoring Department, Swiss Federal Veterinary Office, Schwarzenburgstrasse 155, 3003 Bern, Switzerland

## Abstract

A case of pulmonary tuberculosis caused by *Mycobacterium tuberculosis* was diagnosed in a horse. Clinical evaluation performed prior to euthanasia did not suggest tuberculosis, but postmortem examination provided pathological and bacteriological evidence of mycobacteriosis. In the lungs, multiple tuberculoid granulomas communicating with the bronchiolar lumen, pleural effusion, and a granulomatous lymphadenitis involving mediastinal and tracheobronchial lymph nodes were found. Serologic response to *M. tuberculosis* antigens was detected in the infected horse, but not in the group of 42 potentially exposed animals (18 horses, 14 alpacas, 6 donkeys, and 4 dogs) which showed no signs of disease. Diagnosis of tuberculosis in live horses remains extremely difficult. Four of 20 animal handlers at the farm were positive for tuberculous infection upon follow-up testing by interferon-gamma release assay, indicating a possibility of interspecies transmission of *M. tuberculosis*.

## 1. Introduction

Tuberculosis (TB) is a zoonotic disease caused by *Mycobacterium tuberculosis, M. bovis,* or other members of the *M. tuberculosis* complex in a broad range of mammal hosts [1, 2]. Although natural susceptibility to infection may vary among humans and animals, interspecies transmission is not uncommon, especially in captive wildlife populations in which multi-host TB outbreaks have been reported [3, 4]. Horses are believed to be more resistant to mycobacterial infections compared to other livestock species [5–8]. The current incidence of TB in horses is extremely low, especially in countries with established control programs [9]. Nevertheless, equine cases of clinical disease due to *M. bovis*, *M. avium*, or *M. tuberculosis* have been described [7, 10–12]. Among the tuberculous mycobacteria, *M. bovis* has historically been the predominant causative agent, whereas *M. tuberculosis* is less commonly isolated from horses [9, 13]. However, effective bovine TB control programs in many countries have further reduced the incidence of *M. bovis* infection in horses over the last 20 years. Recently, an isolated case of TB due to *M. bovis* was reported by Keck et al. [14] in a horse living in close contact with infected cattle in the Camargue region of France, an area known for *M. bovis* infection in fighting bulls [15].

Diagnosis of mycobacterial infections in horses is confounded by the atypical morphology of lesions, diverse clinical signs of infection, and limited options available for antemortem testing [9]. The most frequent clinical signs are lethargy and chronic weight loss. Terminal signs of pulmonary TB in horses are fever, dyspnea, and cough. In most countries, tuberculin skin test is not recommended due to its poor accuracy in this species [4, 16]. Definitive diagnosis of equine TB relies on postmortem examination with histopathological assessment of affected tissues including acid fast staining and PCR for *IS 6610* (which identifies *M. tuberculosis* complex organisms) and culture for *Mycobacterial *spp.. Currently, no blood-based TB tests are described for use in horses.

Several antibody assay platforms using recombinant proteins of *M. tuberculosis* or *M. bovis* have recently been developed for rapid TB detection in multiple-host species including free-ranging and captive wildlife as well as in domestic animals [17–21]. As these assays have shown potential for use in a wide range of species (e.g., cattle, deer, camelids, elephants, badgers, wild boar, etc.), they may also be useful for antemortem TB diagnosis in horses. The present report describes a case of pulmonary TB caused by *M. tuberculosis* in a horse, the ensuing investigation of exposed animals and animal handlers at the farm, and evaluation of serologic response in the index case.

## 2. Materials and Methods

### 2.1. Animals

The index case was a 20-year-old Romanian Warmblood gelding (Orlov horse) imported from Poland to Switzerland in 1993. Fourteen other horses were cohoused with the index case within a stable. Fifty-eight animals including 14 alpacas, 6 donkeys, 29 goats, 5 sheep, and 4 horses lived on a pasture adjacent to the horse stable and were all taken care of by the same staff. Four dogs had free access to the stable and the pasture. In addition, 5 horses from known TB-free area in the United States were included in the study as a negative control group for serology evaluation.

### 2.2. Identification of M. tuberculosis

Mycobacteria were identified by PCR on formalin-fixed, paraffin-embedded tissue samples of lung and pulmonary lymph nodes as previously reported [22]. DNA was extracted, and spoligotyping was performed as described [23]. The data was compiled in Microsoft Excel and analyzed by using the MIRU-VNTR plus online analysis tool (http://www.miru-vntrplus.org) [24]. Differentiation of the *M. tuberculosis* complex members was carried out by the GenoType 1 MTBC assay (Hain Lifescience GmbH, Nehren, Germany).

### 2.3. Tuberculin Skin Test (TST)

Goats and sheep were tested by single cervical tuberculin skin test, which was performed by intradermal injection of 2000 IU of bovine purified protein derivative (Bovituber, Synbiotics Europe, Lyon, France).

### 2.4. Serology

Three serological tests developed for rapid detection of TB in various host species included VetTB STAT-PAK, multiantigen print immunoassay (MAPIA), and dual path platform (DPP) VetTB test (Chembio Diagnostic Systems, Inc., Medford, NY, USA). The immunoassay procedures were performed using animal serum samples in accordance with the manufacturer's instructions as previously described [19]. VetTB STAT-PAK kit (also known as rapid test (RT)) and DPP VetTB assay used several recombinant antigens of *M. tuberculosis* or *M. bovis* including ESAT-6, CFP10, and MPB83 protein. Additionally, we applied lipoarabinomannan (LAM) for antibody detection in a DPP assay. MAPIA employed a panel of 14 mycobacterial antigens [20] to detect serum IgG antibody using peroxidase-labeled streptococcal protein G (Sigma, St. Louis, MO, USA) diluted 1 : 1000 or to detect serum IgM antibody using peroxidase-conjugated antibody to horse IgM (Kirkegaard Perry Laboratories, Gaithersburg, MD USA) diluted 1 : 1000, along with substrate 3,3′,5,5′-tetramethylbenzidine (Kirkegaard Perry Laboratories, Gaithersburg, MD USA). Test results were read visually and by using a DPP reader device to measure reflectance in relative light units (RLUs) or densitometry to measure intensity of MAPIA bands in arbitrary units as described earlier [19].

### 2.5. Interferon-Gamma Release Assay (IGRA)

The T-SPOT.TB assay (Oxford Immunotec, Oxford, UK) was used in accordance with the manufacturer's recommended procedure for screening of the farm personnel for latent TB infection (LTBI). The employees were additionally tested by chest X-ray to rule out active pulmonary TB. Two months after the euthanasia of the infected horse, a total of 20 persons including the owner and his companion, the veterinarian, 2 pathologists, 7 workers in the stable, and 8 employees of the farrier were evaluated. Five subjects originated from countries with moderate incidence of human TB (3 from Bosnia, 1 from Portugal, 1 from Turkey), whereas 15 subjects were from low-incidence countries (11 Swiss, 2 French, 2 Italian). Eighteen individuals were evaluated by chest X-ray (all employees, except for the 2 pathologists), and 17 persons had the T-SPOT.TB assay. The IGRA testing was not performed for the 3 oldest subjects (aged 65, 79, and 93) because of the low sensitivity demonstrated for this test in senior population [25] and generally higher probability of harboring previously acquired infection [26]. Subjects with a positive test result had a medical visit including history interview and clinical examination.

## 3. Results and Discussion

### 3.1. Clinical Findings

The index case, a 20-year-old Romanian Warmblood gelding, presenting with symptoms of cardiac insufficiency and ventral edema in July 2010 was referred to the Equine Clinic of the University of Berne with a suspicion of pericarditis and signs of azotemia. Prior to referral, the horse had been febrile (40.0°C) and showed edema in the pectoral and ventral regions for 4 days. The gelding had been treated by the referring veterinarian with antibiotics and nonsteroidal anti-inflammatory drugs. Upon presentation to the Equine Clinic of the University of Berne, the horse was depressed with a heart rate of 60 beats per minute, a respiratory rate of 40 breaths per minute, and a rectal temperature of 38.3°C. The mucous membranes were moist and slightly red with a capillary refill time of 2s. The complete blood count revealed a packed cell volume of 39%, a clear hyperproteinemia of 80 g/L, a marked leucocytosis (33 × 10^3^/*μ*L), and eosinophilia (18.3 × 10^3^/*μ*L). Fibrinogen was within normal range at 2 g/L. Urea and creatinine were well above referenced range with 18 mmol/L and 233 *μ*mol/L, respectively. A thoracic ultrasound examination was performed and revealed severe bilateral pleural and pericardial effusion. Measured lactate in the drained pleural effusate was not different to serum lactate levels. A pericardiocentesis was not performed. Radiographs, acquired after draining the pleural effusion, showed a dense bronchiointerstitial pattern. No abnormalities of the esophagus or stomach were detected on gastroscopy. The clinical differential diagnoses included intra-thoracicneoplasia and infectious pneumonia with pleuritis. Twenty four hours later, the clinical condition deteriorated rapidly with increasing tachypnea (respiratory rate of 40 breaths per minute) and quickly recurring pleural effusion. No etiological diagnosis had been made at this point; yet due to the deteriorating clinical progression and a guarded prognosis, the owner elected to have the horse euthanized.

### 3.2. Necropsy Findings

Tan firm nodules ranging from 2 to 3 mm were disseminated throughout the pulmonary parenchyma. The tracheobronchial and mediastinal lymph nodes were moderately enlarged, with irregular, often multinodular surface. On cut surface the lymph nodes showed multiple nodules with a necrotic core. A severe acute fibrinous pericarditis and an acute to subacute fibrinous pleuritis with abundant pleural exudation and multiple pleuro-diaphragmatic and pleuro-pericardial adherences were also present. Additional findings included mild diffuse subcutaneous edema, endocardial fibroelastosis and acute ulcers along the margoplicatus of the squamous portions of the gastric mucosa.

**Figure 1:**
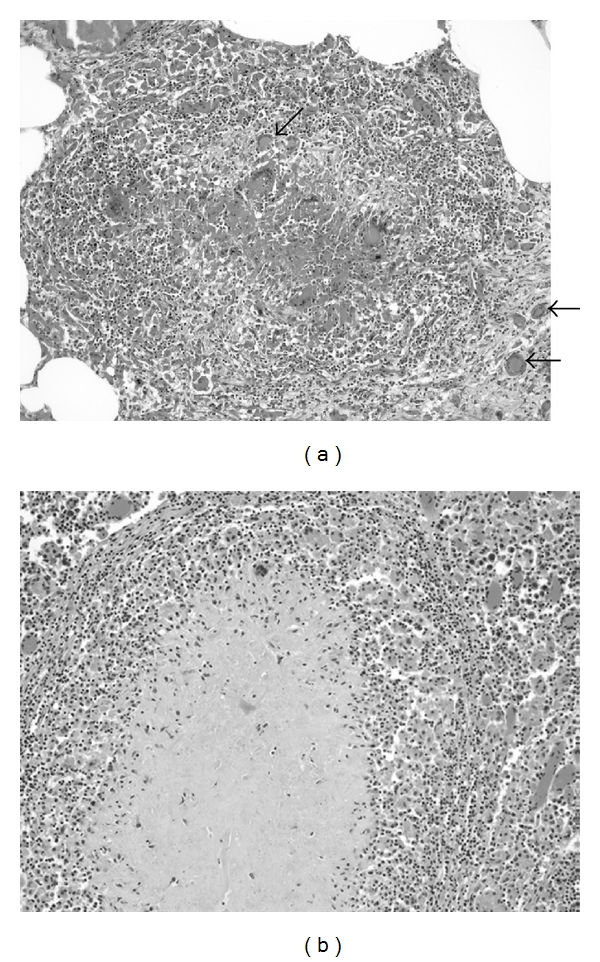
Histology of lung (a) and pulmonary lymph node (b) lesions. Typical tuberculoid granulomas demonstrating central necrosis (particularly apparent in the lymph node lesion) surrounded by epithelioid macrophages and multinucleated, Langhans-type giant cells (examples noted with arrows) admixed with lymphocytes.

Histologically, typical tuberculoid granulomas with central necrosis surrounded by epitheliod macrophages and multinucleated, Langhans-type giant cells admixed with few lymphocytes were detected in the lung and pulmonary lymph nodes (Figure 1). Some granulomas were located within the walls of bronchioli. Multiple granulomas showed marked fibrosis admixed with few multinucleated giant cells. Ziehl-Neelsen and Fite-Faraco stains of multiple granulomas in the lung and lymph nodes revealed no acid fast bacilli. Based on these findings, a presumptive diagnosis of a pulmonary mycobacteriosis caused by organisms of the *M. tuberculosis* complex was made.

Using formalin-fixed and paraffin-embedded tissue samples of lungs and lymph nodes, *M. tuberculosis* was identified by PCR amplification of DNA extracts. Culture isolation was not attempted, as the specimens collected at necropsy were all processed for histology. Spoligotyping revealed a member of the *M. tuberculosis* complex exhibiting an orphan octal code (575247437763771).

Thus, results of postmortem examination of the horse revealed the presence of miliary TB with pleural effusion and necrotic lymph nodes, some of which had eroded into the bronchi.Earlier studies (reviewed in [9, 13]) indicate that most TB cases in horses are due to *M. bovis* and the most common site of infection is the gastro-intestinal tract, suggesting ingestion (oral route) as a major way of transmission in this host species. In contrast, we found disease due to *M. tuberculosis* predominantly localized in the thoracic organs. This feature combined with the observation that the infected horse was coughing for at least two days before death emphasizes the zoonotic concerns associated with this case.

### 3.3. Antibody Response

**Figure 2:**
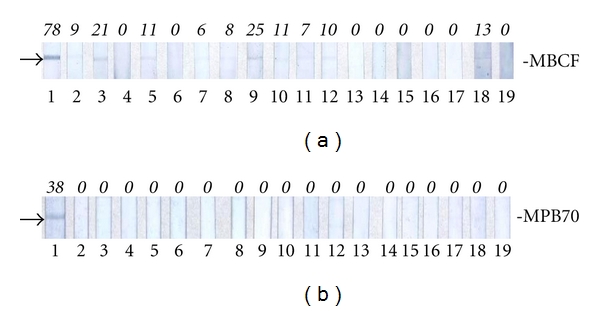
IgG (a) and IgM (b) antibody responses of the horse infected with *M. tuberculosis.* MAPIA was performed as described in the Materials and Methods Section. Strip images represent individual results obtained with serum samples from the infected horse (no. 1), and 18 presumed non-infected horses from the same farm (nos. 2–19). Arrows point at the bands indicating antibody reactivity found in the infected horse. Printed antigens are shown on the right. Figures in italic above the strips indicate MAPIA densitometry values (in arbitrary units) obtained for respective serum samples.

A serum sample collected from the index case at the time of euthanasia was tested by RT, MAPIA, and DPP assays with various antigens. The *M. tuberculosis*-infected horse was nonreactive by RT or DPP VetTB assay, thus suggesting the absence of detectable antibodies to ESAT-6, CFP10, and/or MPB83 in the serum. However, MAPIA revealed IgM antibody to MPB70 protein and strong IgG reactivity to *M. bovis *culture filtrate (MBCF), whereas no IgM against MPB70 and only borderline IgG binding to MBCF were found in sera from 18 potentially exposed but presumed noninfected horses from the farm (Figure 2) or among 5 negative control horses from the United States (data not shown). Other antigens used in MAPIA elicited no detectable antibody response in the infected animal or provided no discriminating power to distinguish the index case from the other horses. Further, using LAM of *M. tuberculosis* as a test antigen in DPP assay format allowed for detection of a strong serum IgG reactivity in the infected horse (DPP reader reflectance = 235 RLU) if compared to the control group (5 ± 12 RLU, *n* = 5) or to the potentially exposed but presumed non-infected horses (8 ± 14 RLU, *n* = 18). This observation is consistent with the MAPIA IgG reactivity to MBCF, presumably because this crude antigen preparation contains LAM which is known to elicit particularly robust antibody responses in humans and animals diagnosed with TB (our unpublished observations).

Thus, the infected horse developed a serologic response to certain *M. tuberculosis* antigens, such as LAM, MPB70, or MBCF. The antibody reactivity involved both IgM and IgG classes, as has also been shown for cattle experimentally infected with *M. bovis* [17]. Antigens recognized by the horse serum did not include the key specific targets identified in previous studies for other host species, such as ESAT-6, CFP10, or MPB83 protein [18]. As a result, the available animal-side assays employing the latter antigens and designed for those species failed to detect specific antibody in serum from the infected horse. The results illustrate the view that, when developing novel TB immunodiagnostics for various hosts, it is crucial to validate the choice of diagnostically important antigens being selected for each new species individually. Finding the antibody response in the M. tuberculosis-infected horse in the present study encourages further efforts to identify suitable antibody-reactive antigens for developing accurate TB serodiagnostics for this species. 

### 3.4. Testing Exposed Animals

 Fourteen horses lived in the same stable as the *M. tuberculosis*-infected horse. Other horses (*n* = 4), goats (*n* = 29), sheep (*n* = 5), donkeys (*n* = 6), and alpacas (*n* = 14) lived on a pasture adjacent to the horse stable and were taken care of by the same staff. Four dogs had free access to the stable and the pasture. These animals were considered exposed, as they had been in direct or indirect contact with the *M. tuberculosis*-infected horse. TST was negative in all goats and sheep. No TST was performed in horses, donkeys, alpacas, and dogs, because no validated protocols were available for these species. Serological testing with RT, DPP VetTB, and MAPIA of the alpacas, donkeys, and dogs showed no antibody reactors. To date, none of these animals has developed clinical symptoms that would be consistent with TB.

### 3.5. Testing Farm Personnel

Screening for LTBI was performed by the T-SPOT.TB assay, in accordance with the United States (Center for Disease Control and Prevention) and Swiss (Federal Bureau for Public Health) recommendations. The farm owner and the employees had to be tested for two objectives: (1) to search for a possible source of infection among the “care givers” of the infected horse (employees of the stable), since animal-to-animal transmission was highly unlikely, and (2) to search for subjects who could have been infected by the horse that had reportedly been coughing at least 2 days prior to death.

Among the 18 employees evaluated by chest X-ray, no indication for active TB was found (Table 1). One Swiss person showed a pulmonary nodule that was further investigated by the patient's general practitioner and was then presumed to be of neoplastic nature. For the 17 persons tested with the T-SPOT.TB assay, 12 results were negative, 4 were positive, and one was indeterminate (twice). Among the 4 IGRA-positive subjects, two originated from countries with moderate incidence of TB (Bosnia and Turkey). According to the history interview records, all 4 had definite or probable previous exposures to TB, as well as variable periods of recent direct or indirect contact with the infected horse. Treatment for LTBI was proposed to all patients with a positive IGRA result (one person refused treatment). To date, none of the tested employees has developed any signs of active TB.

### 3.6. Zoonotic Aspect

 In contrast to *M. bovis* known to affect a wide range of natural hosts, susceptibility to *M. tuberculosis* remains not well defined for many mammal species [1, 2, 4] including horses and others (alpacas, donkeys, goats, sheep, and dogs) potentially exposed to the index case in the present study. Since no signs of infection were found in any of the remaining animals of the six species at the farm, it appeared unlikely that the equine TB case had resulted from animal-to-animal transmission of *M. tuberculosis*. Further, because the isolate was *M. tuberculosis*, not *M. bovis*, one could speculate that the horse infection had been acquired from a human case of active TB but not necessarily from any of the employees with a positive IGRA result. Moreover, an alternative scenario cannot be ruled out that LTBI detected in certain personnel during the follow-up investigation could have resulted from exposure to the *M. tuberculosis*-shedding horse. The IGRA-positive subjects have reportedly been exposed to TB in the past, but no earlier records on routine testing (TST or IGRA) were available to determine their pre-existing infection status. Thus, the origin of *M. tuberculosis* infection of the horse described herein remains unknown.

## 4. Conclusions

A rare case of TB due to *M. tuberculosis *was found in a horse. Based on the clinical evaluation results, TB was not suspected in this animal. However, postmortem examination by pathology and molecular methods led to diagnosis of pulmonary disease caused by *M. tuberculosis*. Antemortem testing of horses for TB remains challenging, because traditional methods, such as TST, have not been validated for this species. An antibody response to *M. tuberculosis* antigens was found in the infected horse, but not in the exposed animals that had no signs of disease. Follow-up testing of personnel involved in taking care of these animals revealed 4 IGRA-positive results indicating LTBI in those subjects. The findings suggest a possibility of interspecies transmission of *M. tuberculosis, *although the infection source remains unclear.

## Figures and Tables

**Table 1:** Human contact testing results.

Group	Gender^a^	Age, years	Country of origin	Chest X-ray	T-SPOT.TB
	M	93	Italy	Neg	ND^b^
Owner, companion, veterinarian, and pathologists	F	45	Switzerland	Neg	Neg
	M	61	Switzerland	Neg	Pos
	M	50	Switzerland	ND	Neg
	F	32	Italy	ND	Neg

Workers in stable	M	65	Switzerland	Neg	Indeterminate^c^
	F	65	Switzerland	Neg	ND
	M	48	Portugal	Neg	Neg
	M	79	Switzerland	Nodule, non-TB	ND
	M	57	Bosnia	Neg	Pos
	F	55	Bosnia	Neg	Neg
	M	58	Bosnia	Neg	Neg

Farriers	M	19	Switzerland	Neg	Neg
	M	55	Switzerland	Neg	Neg
	M	21	Switzerland	Neg	Neg
	F	23	Switzerland	Neg	Neg
	M	26	Switzerland	Neg	Neg
	M	44	France	Neg	Pos
	M	26	France	Neg	Neg
	M	51	Turkey	Neg	Pos

^a^M, male; F, female

^b^ND, not done

^c^Tested indeterminate repeatedly.
